# NADPH Oxidase Gene Polymorphism is Associated with Mortality and Cardiovascular Events in 7-Year Follow-Up

**DOI:** 10.3390/jcm9051475

**Published:** 2020-05-14

**Authors:** Milena Racis, Wojciech Sobiczewski, Anna Stanisławska-Sachadyn, Marcin Wirtwein, Elżbieta Bluj, Michał Nedoszytko, Joanna Borzyszkowska, Janusz Limon, Andrzej Rynkiewicz, Marcin Gruchała

**Affiliations:** 1 First Department of Cardiology, Medical University of Gdańsk, ul. Dębinki 7, 80-211 Gdańsk, Poland; wsob@gumed.edu.pl (W.S.); ebluj@gumed.edu.pl (E.B.); nedo@gumed.edu.pl (M.N.); mgruch@gumed.edu.pl (M.G.); 2 Department of Biology and Genetics, Medical University of Gdańsk, ul. Dębinki 1, 80-211 Gdańsk, Poland; atanasiu@gumed.edu.pl (J.B.); jlimon@gumed.edu.pl (J.L.); 3 Department of Molecular Biotechnology and Microbiology, Gdańsk University of Technology, ul. Narutowicza 11/12, 80-233 Gdańsk, Poland; anna.stanislawska@pg.edu.pl; 4 Department of Pharmacology, Medical University of Gdańsk, ul. Dębinki 7, 80-211 Gdańsk, Poland; marcin.wirtwein@gumed.edu.pl; 5 Department of Cardiology and Cardiosurgery, University of Warmia and Mazury in Olsztyn, Al. Warszawska 30, 10-082 Olsztyn, Poland; andrzej.rynkiewicz@uwum.edu.pl

**Keywords:** NADPH oxidase, *CYBA*, polymorphism, atherosclerosis, CAD, cardiovascular disease

## Abstract

The *CYBA* gene encodes the regulatory subunit of NADPH oxidase, which maintains the redox state within cells and in the blood vessels. That led us to investigate the course of coronary artery disease (CAD) with regards to *CYBA* polymorphisms. Thus, we recruited 1197 subjects with coronary atherosclerosis and observed them during 7-year follow-up. Three *CYBA* polymorphisms: c.214C>T (rs4673), c.-932G>A (rs9932581), and c.*24G>A (1049255) were studied for an association with death, major adverse cardiovascular events (MACE) and an elective percutaneous coronary intervention or coronary artery bypass grafting (PCI/CABG). We found an association between the *CYBA* c.214C>T polymorphism and two end points: death and PCI/CABG. *CYBA* c.214TT genotype was associated with a lower risk of death than C allele (9.5% vs. 21%, *p* < 0.05) and a higher risk of PCI/CABG than C allele (69.3% vs. 51.7%, *p* < 0.01). This suggests that the *CYBA* c.214TT genotype may be a protective factor against death OR = 0.47 (95%CI 0.28–0.82; *p* < 0.01), while also being a risk factor for an elective PCI/CABG OR = 2.36 (95%CI 1.15–4.82; *p* < 0.05). Thus, we hypothesize that among patients with coronary atherosclerosis, the *CYBA* c.214TT genotype contributes to atherosclerotic plaque stability by altering the course of CAD towards chronic coronary syndrome, thereby lowering the incidence of fatal CAD-related events.

## 1. Introduction

NADPH oxidase is an enzymatic complex, since it consists of several subunits and, according to functional studies, it serves as a principal source of reactive oxygen species (ROS) within the organism [1,2]. The upregulation of NADPH oxidase may significantly contribute to increased ROS production since it causes imbalance between pro-oxidants and antioxidant agents and can thus be the cause of oxidative stress [3,4]. In the context of cardiovascular disease, a genetic polymorphism of NADPH oxidase seems to be vital since ROS overproduction, associated with inflammation and metabolic dysfunction, may contribute to atherosclerotic plaque instability and by that means be a trigger of abrupt manifestation of cardiovascular disease [5,6,7]. Oxidative stress seems to be the main factor in the atherosclerotic plaque formation and its further modification [8,9]. 

P22phox (often referred to as cytochrome b-245) is one of the main subunits of NADPH oxidase and, as it is considered regulatory, contains the entire electron transport apparatus of NADPH oxidase and thereby acts as a physical conduit for transport of electrons across the membrane. Some in vitro studies have revealed the essential role of this subunit on NADPH oxidase regulation and stability, where si-RNA-mediated p22Phox downregulation leads to decreased function of NADPH oxidase [10]. This is why the genetic polymorphism of the *CYBA* gene, encoding p22phox, has been of a special interest over almost two decades, especially in the context of cardiovascular disease. Given that a significant number of polymorphisms has been reported within the *CYBA* gene, we can assume that its genetic variability may play a vital role in the oxidative stress formation triggered by the NADPH oxidase [11,12,13,14,15].

Our interest focused on three *CYBA* polymorphisms which to our best knowledge are the most significant in the context of cardiovascular disease: the *CYBA* c.214C>T (often referred to as *CYBA* 242T>C) (rs4673) in the 214 position in the exon 4 (substitutes histidine (His) by tyrosine (Tyr) in the potential heme-binding sites), the *CYBA* c.*24G>A (often referred to as *CYBA* 640A>G) (rs1049255) localized in the 3′untranslated region and the *CYBA* c.-932G>A (often referred to as *CYBA*-930G>A) (rs9932581) in the gene promoter region.

The aim of the study was to investigate the association between the *CYBA* c.214C>T, *CYBA* c.*24G>A, and *CYBA* c.-932G>A polymorphisms and the course of CAD in a large population comprising 1,197 subjects from northern Poland. Specifically, the following evaluating end points were considered: (1) death (all-cause mortality); (2) major adverse cardiovascular events (MACE): death, acute coronary syndromes (ACS) or stroke; and (3) elective percutaneous coronary intervention and/or coronary artery bypass grafting (PCI/CABG).

## 2. Materials and Methods

### 2.1. Study Population

The present study was based on a database containing health-related data collected in the framework of the project conducted in the First Department of Cardiology of the Medical University of Gdansk between August 2003 and August 2006. The database, which was previously used in other genetic and non-genetic studies [16,17] contains clinical data and coronary angiography results of 1908 Caucasian patients consecutively admitted to the clinic with typical symptoms of myocardial angina or positive results of non-invasive diagnostic tests, who were referred for diagnostic coronary angiography. On admission, the interview concerning history of cardiovascular disease, co-existing diseases and risk factors was taken. Hypertension was diagnosed on the basis of measurements during hospitalization or the patient was on hypotensive therapy. Diabetes mellitus was diagnosed on the basis of fasting glucose levels, oral glucose tolerance test or use of hypoglycemic therapy. Smoking status was recognized on an interview alone. On the admission day, after obtaining patient’s informed consent, fasting blood samples were collected in order to measure total cholesterol, low-density lipoprotein cholesterol, high-density lipoprotein cholesterol, triglycerides, glucose and other biochemical parameters. Additionally, blood samples were collected in order to isolate DNA from peripheral blood lymphocytes. 

After considering the inclusion and exclusion criteria, 1197 individuals out of 1908 were recruited into the present study. Inclusion criteria were: age of at least 18 years at the moment of recruitment, presence of one or more atherosclerotic lesions confirmed in the coronary angiography and complete molecular data regarding three polymorphic sites investigated within this study. Exclusion criteria were: lack of coronary atherosclerotic lesions and missing molecular data of at least one of genetic polymorphisms. The protocol of this study was approved by the Ethics Committee of the Medical University of Gdansk (ref no. NKEBN/138/2008 issued on 15 May 2008) and conforms to the ethical guidelines of the 1964 Declaration of Helsinki and its later amendments.

### 2.2. Assessment of Coronary Atherosclerosis

In the Department of Invasive Cardiology coronary angiography was performed in order to assess the extent of atherosclerosis in coronary arteries and the need for invasive treatment. The prevalence, distribution, and extent of the atherosclerotic lesions were evaluated by at least two independent invasive cardiologists. In order to be enrolled into the study, the patient must have had atherosclerosis confirmed in the coronary angiography, independently of the clinical significance of the lesion and need for invasive treatment. Every atherosclerotic lesion with 30–100% of lumen narrowing was considered as a presence of atherosclerosis in the coronary arteries.

### 2.3. Genetic Analyses

DNA was isolated from venous blood lymphocytes. DNA quality was examined using NanoDrop spectrophotometer (Thermo Scientific). The *CYBA* c.214C>T polymorphism was detected by PCR restriction fragment length polymorphism (RFLP) assay. Individual PCR amplification reactions (25 μL) were composed of 2 μL sample DNA (at an average concentration: 30 ng/µL), 2.5 μL reaction buffer (10×), 2 mM MgCl_2_, dNTPs (2 mM each), 0.5 U Taq DNA polymerase (Applied Biosystems), 0.5 μM forward primer (5′-TGCTTGTGGGTAAACCAAGG-3′) and 0.5 μM reverse primer (5′-CACTTACCTCAGTGTTTTTCC-3′). PCR was performed with an initial incubation at 95 °C for 5 min followed by 35 cycles of denaturation at 95 °C for 30 s, annealing at 55 °C for 30 sec, and extension at 72 °C for 30 s. The 353 bp (base pair) product was subsequently digested with a restriction enzyme *RsaI* (Fermentas) which resulted in fragments: 160 bp and 193 bp for the TT genotype, 160 bp, 193 bp and 353 bp for the CT genotype, and 353 bp for the CC genotype. All ambiguous samples were genotyped a second time.

The *CYBA* c.*24G>A polymorphism was determined using a TaqMan 5′nuclease real-time PCR assay (LightCycler 480 II, Roche Molecular Diagnostic Inc., CA, USA). Individual PCR amplification reactions (20 μL) were composed of 2 μL sample DNA, 1× LightCycler 480 Probes Master (Roche), 0.5 μM forward primer (5′-CCCATCCCGGTGACCGACGA–3′) and 0.5 μM reverse primer (5′-CAGGCCTCGGGAACCATCGC-3′), 50 nM ‘G’-specific probe (FAM-TGCCCTCCCGCCAGGTGC-BHQ1), and 50 nM ‘A’-specific probe (HEX-TGCCCTCCCACCAGGTGCA- BHQ1). PCR was performed with an initial incubation at 95 °C for 10 min followed by 60 cycles of denaturation at 95 °C for 15 s, extension/5′ nuclease step at 66 °C for 15 s and elongation at 72 °C for 30 s. Dual fluorescence was detected after each completed cycle. Each sample was analyzed in duplicate.

The *CYBA* c.-932G>A polymorphism was determined using a TaqMan 5′nuclease real-time PCR assay (LightCycler 480 II, Roche Molecular Diagnostic Inc., CA, USA). Individual PCR amplification reactions (20 μL) were composed of 2 μL sample DNA, 1× LightCycler 480 Probes Master (Roche), 0.5 μM forward primer (5′-CTGGAATGGTGGCAGGAGT-3′) and 0.5 μM reverse primer (5′- CGGGATGCTGGTTTACGAA-3′), 50 nM ‘A’-specific probe FAM-GGCAGTAATGCTGGT-BHQ1), and 50 nM ‘G’-specific probe, and (HEX-GGCAGCAATGCTGGT-BHQ1). PCR was performed with an initial incubation at 95 °C for 10 min followed by 60 cycles of denaturation at 95 °C for 30 s and extension/5′ nuclease step at 56 °C for 1 min. Dual fluorescence was detected after each completed cycle, and each sample was analyzed in duplicate. The validation of the results by sequencing exemplary PCR products of all the three *CYBA* polymorphisms was performed. 

Positive controls representing all genotype classes (both homozygotes and heterozygote) and a negative control were included in each plate. Samples were genotyped in duplicate.

### 2.4. Follow-Up Study

All subjects were observed from the date of coronary angiography until 31 December 2011. The prospective data determining evaluated end points were collected in 7-year follow-up (mean of 90 months) and were obtained from the National Polish Health Service by means of the patients’ name and the Polish residence identification number (PESEL). 

The following end points were obtained: (1) all-cause mortality (cardiovascular and non-cardiovascular causes); (2) MACE—major adverse cardiovascular events: death, acute coronary syndromes (ACS) and strokes; and (3) elective percutaneous coronary intervention (PCI) and/or coronary artery bypass grafting (CABG). The causes of death were coded by the means of International Statistical Classification of Diseases and Related Health Problems. The term acute coronary syndrome (ACS) was applied to non-ST elevation myocardial infarction (NSTEMI), unstable angina (UA), and ST-elevation myocardial infarction (STEMI). ACS and stroke diagnosis was performed according to the guidelines of the European Society of Cardiology and European Stroke Organisation, respectively. 

### 2.5. Statistical Analyses

The main goal of the analysis was to search for a relationship between the *CYBA* genotypes and overall survival or survival to an end point in 7-year follow-up. Kaplan–Meier analysis was used for this purpose. The crude survival probabilities were plotted using log rank test.

The prevalence of the evaluated end points was analyzed between the carriers of all the genotypes, subsequently associations were tested using genetic models: dominant (carriers of at least one risk allele vs. the rest) and recessive (carriers of two risk alleles vs. the rest). This created the following groups for analyses in the dominant model: *CYBA* c.-932G allele (GG and AG genotypes) vs. AA genotype carriers, *CYBA* c.214T allele (TT and CT genotypes) vs. CC genotype carriers, *CYBA* c.*24G allele (GG and AG genotypes) vs. AA genotype carriers. The groups for the analyses in recessive model were: *CYBA* c.-932GG genotype vs. A allele (AG and AA genotypes) carriers, *CYBA* c.214TT genotype vs. C allele (CT and CC genotypes) carriers, *CYBA* c.*24GG genotype vs. G allele (AG and AA genotypes) carriers.

The time durations to the three evaluated endpoints were determined from the baseline date to the date of these events (observations complete) or—when without an end-point—to the end of the observation (observations censored). Regarding combined endpoints (MACE, PCI/CABG), the first event in each category was only considered in the statistical analyses. Whereas considering the PCI/CABG endpoint, the subjects who died during the study without an elective PCI/CABG were treated as observations censored. The relative hazard ratio (HR) and 95%CI were estimated with adjustment for possible confounding clinical variables using COX proportional hazard regression model. Clinical characteristics of the patients were presented as means ± SD (standard deviation) for continuous variables (i.e., BMI, cholesterol level) or as percentages for categorical variables (i.e., smoking status, hypertension, diabetes). Smoking status was self-reported.

Deviations from the Hardy–Weinberg equilibrium for the genotypes were assessed by chi square test. The chi square tests were used to compare frequencies of categorical variables between groups, the Student’s *t*-test or the U-test were used to compare levels of continuous variables between groups.

The level of statistical significance was set at *p* < 0.05. Statistical analyses were performed using Statistica version 10 (StatSoft Inc. Tulsa, OK, USA).

## 3. Results

The 7-year follow-up of the initial study group of 1,197 patients reported 203 incidence of death, 425 cases of MACE (death, ACS, or stroke), and 624 cases of PCI and/or CABG.

The patients who died during the follow-up study were statistically older than those who survived. The group of patients where MACE occurred was characterized by an older age and also a higher prevalence of hypertension, diabetes, and higher level of triglycerides. Those individuals who underwent PCI/CABG had higher BMI and higher level of fasting glucose but lower level of HDL-cholesterol and lower percent of an actual smoking status (however, the percent of individuals with a history of smoking tended to be higher in this group). The detailed clinical characteristics and the prevalence of the risk factors in the particular subgroups with regards to the evaluated end points are presented in Table 1.

Our analyses showed clinical significance of only one examined polymorphism: *CYBA* c.214C>T, which proved to have meaning regarding two out of three studied endpoints: death and PCI/CABG. As a result, no association of *CYBA* gene polymorphisms with MACE (death, ACS or stroke) was found whatsoever. Furthermore, no statistically significant outcome considering *CYBA* c.*24G>A and *CYBA* c.-932G>A polymorphisms was observed. An association between the *CYBA* c.214C>T polymorphism and death and PCI/CABG was found in analyses performed according to the recessive model: in the group defined by the TT genotype in comparison with the C allele group there was a significantly lower prevalence of death and significantly higher prevalence of PCI/CABG (Table 2).

**Figure 1:**
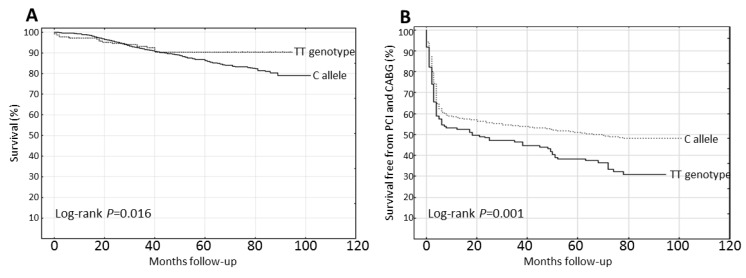
7-year Kaplan–Meier curves for crude survival within the recessive model of the *CYBA* c.214C>T polymorphism: (**A**) Significantly lower risk of death (higher probability of overall survival) among the subjects with the TT genotype in comparison to C allele carriers. (**B**) Significantly higher risk of undergoing PCI and/or CABG among the subjects with the TT genotype in comparison to C allele carriers. PCI: percutaneous coronary intervention; CABG: coronary artery bypass grafting.

The Kaplan–Meier analysis with a comparison of the Crude Survival probabilities showed that the probability of overall survival was statistically higher in the group with the TT genotype (90.5%) in comparison to the C allele carriers (79%), while the risk of death was 9.5% and 21%, respectively (*p* < 0.05) (Figure 1A). On the contrary, the probability of survival free from PCI/CABG within the *CYBA* c.214C>T polymorphism was statistically lower among the subjects who carried the TT genotype (30.7%) in comparison to those who carried the C allele (48.3%), and the risk of undergoing PCI/CABG was 69.3% and 51.7%, respectively (*p* < 0.01) (Figure 1B).

Furthermore, in the COX proportional hazard regression model the *CYBA* c.214TT genotype was defined among other clinical factors as an independent protective factor of all-cause mortality (HR) (OR = 0.47(95%CI 0.28–0.82; *p* < 0.01) and as an independent risk factor of the elective PCI/CABG (OR = 2.04 (95%CI 1.18–3.52; *p* < 0.05).

## 4. Discussion

The analyses indicate that among patients with coronary atherosclerosis, the risk of death is significantly lower and the risk of undergoing elective PCI and/or CABG is significantly higher in subjects carrying the *CYBA* c.214TT genotype when compared to those carrying the *CYBA* c.214C allele. Thus we suppose that the *CYBA* c.214TT genotype is responsible for a slow growth of an atherosclerotic plaque (stable coronary syndromes treated with elective PCI or CABG), whereas the other variant—CYBA c.214C allele makes the atherosclerotic lesion more unstable which can lead to plaque rupture (increased incidence of ACS complicated with death).

These results may seem contradictory, but they are in accordance with a pathophysiological background that makes a clear distinction between stable plaques responsible for stable coronary syndromes and unstable plaques leading to ACS. Already in 1985, Davies and Thomas stated that acute coronary syndromes are not usually the result of a gradual narrowing of the coronary artery lumen, but rather the result of a sudden disruption of an atherosclerotic lesion that did not cause critical narrowing prior to the incident [18]. These findings have been confirmed in subsequent research [19,20,21].

**Figure 2:**
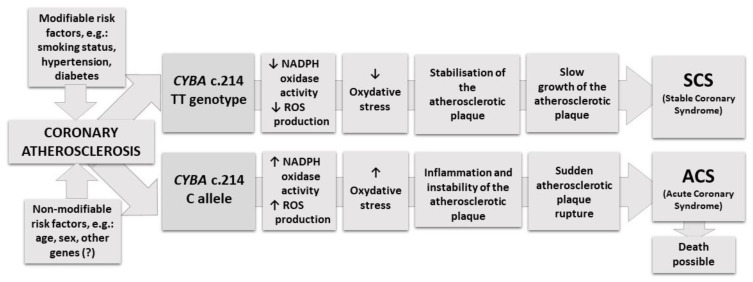
Schematic illustration of hypothesis of how the *CYBA* c.214C>T polymorphism might modulate plaque stability and influence the course of coronary atherosclerosis.

Assuming that the causes of death in the CAD patient population are mainly acute coronary syndromes and their complications, patients who express NADPH oxidase encoded by the *CYBA* 214TT genotype may have a lower probability of suffering a fatal acute coronary syndrome (ACS). NADPH oxidase activity, determined from superoxide production, has been diminished in peripheral mononuclear cells of hypertensive subjects with the *CYBA* c.214T allele [14], in human blood vessels of T allele carriers with CAD [22], in lymphoblastoid cells of T allele carriers [23], and in neutrophils isolated from subjects with TT genotype [24]. Those findings from functional studies facilitate the interpretation of our results. Decreased NADPH oxidase activity and diminished ROS production can reduce vascular oxidative stress and inflammation, which play a main role in atherosclerotic plaque instability and rupture [25,26,27,28,29]. It is well-known that atherosclerosis is a multifactorial disease with both modifiable and non-modifiable risk factors [30,31,32,33]. Therefore, we can assume that mechanisms underlying chronic atherosclerotic plaque growth differ from pathological processes leading to acute plaque rupture, which may be determined by genetic variants associated with oxidative stress [34]. This suggests that the *CYBA c.*214TT genotype increases atherosclerotic plaque stability by lowering oxidative stress levels. Our hypothesis of how this *CYBA* polymorphism might modulate plaque stability and influence the course of coronary atherosclerosis is schematically represented in Figure 2.

Nonetheless, from our analyses of previous studies investigating the association between *CYBA* gene polymorphisms and cardiovascular disease, it can be seen that the results are not consistent and can even be confusing. Most studies and meta-analyses have reported a protective role of the *CYBA* c.214TT genotype in Asian populations [35,36], while the association between this polymorphism and cardiovascular disease in Caucasian populations has been ambiguous [37,38,39,40,41]. This suggests significant heterogeneity across ethnicities in the modulatory role of *CYBA* polymorphisms in CAD. However, in the majority of these studies, the association between *CYBA* polymorphisms and disease was examined in populations comprising cases and healthy controls, a methodology that is different from that of our study. Moreover, in some studies, patients within control groups were recruited only on the basis of a negative interview towards CAD [12,37,38]. This may be misleading as clinical symptoms do not always correlate with the severity of CAD and atherosclerotic plaques can still be present in asymptomatic individuals.

In this study, we only recruited individuals with previously diagnosed CAD confirmed by coronary angiography for subsequent observation during the 7-year follow up. This makes our study unique compared to that of other researchers. Furthermore, this observation was based on data from the National Polish Health Service, which is considered a reliable and non-biased source of data.

Although we assessed patients with cardiovascular disease, we decided to analyze all-cause mortality instead of only cardiovascular causes of death. Our decision is based on the assumption that in a population with a high cardiovascular risk, deaths that appear to have other causes may in fact be caused by cardiovascular complications. Hence, in our opinion, accounting for all-cause mortality is more reliable and unbiased, although it is also possible that the protective influence of the *CYBA* c.214TT genotype may have an influence in other diseases, which may be due to oxidative stress.

One limitation of the study is the small number of deaths, 14, in the *CYBA* c.214TT subgroup, despite having a large study population. However, it should be noted that in modern times, mortality from cardiovascular causes is much lower due to significant progress in diagnosis and treatment. It is also astonishing that we did not report any association of *CYBA* gene polymorphism with MACE. However, there are some reasons for this as clinical events associated with the pathophysiology of MACE may be both a result of a plaque rupture (cardiovascular death, ST-elevation myocardial infarction) or slow growth of a plaque (some cases of unstable angina or non-ST elevation myocardial infarction). As such, we assert that the results could cancel each other out, thus not giving any statistically significant outcome.

Attempts to understand the causes of oxidative stress at a genetic level mainly involve searching for an association between different polymorphic variants and disease phenotypes. It is understood that this creates a knowledge gap, which can only be filled by subsequent functional studies involving gene expression, transcriptomic analyses, and protein function analyses with target selection based on their ability to influence phenotypes. Although studies like ours do not comprehensively establish the molecular mechanisms of CAD-related gene polymorphisms and oxidative stress, they may be useful for future identification of prognostic biomarkers for patients with coronary atherosclerosis to predict the different manifestations of the disease.

## 5. Conclusions

In our analyses the *CYBA* c.214TT genotype has been found to be a protective factor against death, while also being a risk factor for an elective PCI/CABG. Thus, we conclude that a variant of cytochrome b-245 (P22phox) with Tyr residue at the 72 position in amino-acid chain, encoded by the *CYBA* c.214TT genotype, may, by lowering ROS production, impact atherosclerotic plaque stability and alter the course of CAD towards chronic coronary syndromes.

## Figures and Tables

**Table 1:** Clinical characteristics and prevalence of risk factors of the study groups where the three endpoints (death, MACE, PCI/CABG) occurred and did not occur.

	death (*n* = 203)	without death (*n* = 994)	*p*	MACE (*n* = 425)	without MACE (*n* = 772)	*p*	PCI/CABG (*n* = 624)	without PCI/CABG (*n* = 573)	*p*
**Age (years)**	67.1 ± 9	63.6 ± 9	<0.01	65.6 ± 9	63.3 ± 9	<0.01	63.9 ± 9	64.4 ± 10	ns
**Male (%)**	70	66	ns	67	66.7	ns	53.7	46	ns
**BMI (kg/m^2^)**	27.5 ± 4	28 ± 4	ns	27.8 ± 4	28 ± 4	ns	28.2 ± 4	27.7 ± 4	<0.05
**Total cholesterol (mg/dL)**	209 ± 54	205 ± 53	ns	207 ± 57	206 ± 51	ns	209 ± 53	203 ± 54	ns
**LDL cholesterol (mg/dL)**	123 ± 45	123 ± 56	ns	121 ± 43	124 ± 59	ns	126 ± 45	120 ± 63	ns
**HDL cholesterol (mg/dL)**	55 ± 19	54.9 ± 17	ns	54 ± 17	55.5 ± 18	ns	53.4 ± 13	56.8 ± 22	<0.05
**Triglycerides (mg/dL)**	152 ± 99	147 ± 100	ns	157 ± 110	143 ± 93	<0.05	147.8 ± 90	148 ± 110	ns
**Glucose (mg/dL)**	122 ± 46	118 ± 43	ns	121 ± 46	118 ± 42	ns	122 ± 47	116 ± 39	<0.05
**Hypertension (%)**	83	80	ns	84	78	<0.05	53	47	ns
**Diabetes (%)**	29.6	23	ns	29	22	<0.05	55	45	ns
**Actual smokers (%)**	15.5	14	ns	16	14	ns	43	57	<0.05
**History of smoking (%)**	70.5	67	ns	66	68	ns	53	47	ns
**CAD in family (%)**	48.6	55	ns	53	54.5	ns	55	45	ns

ns: non statistically significant; MACE: major adverse cardiovascular events; PCI: percutaneous coronary intervention; CABG: coronary artery bypass grafting; BMI: body mass index; LDL: low-density lipoprotein cholesterol; HDL: high-density lipoprotein cholesterol; CAD: coronary artery disease.

**Table 2:** Prevalence of the evaluated endpoints in the dominant and the recessive model subgroups with the log-rank test *p*-value.

*CYBA* Gene Polymorphisms (Dominant and Recessive Models)		Patients within the Groups *n* (%)	Death *n* (%)	*p*	MACE *n* (%)	*p*	PCI/CABG *n* (%)	*p*
c.214C>T	T allele vs. CC	693 vs. 504 (58% vs. 42%)	114 vs. 89 (16% vs. 18%)	ns	252 vs. 173 (36% vs. 32%)	ns	361 vs. 263 (52% vs. 49%)	ns
	C allele vs. TT	1050 vs. 147 (88% vs. 12%)	**189 vs. 14** **(18% vs. 9%)**	**<0.05**	381 vs. 44 (36% vs. 30%)	ns	**528 vs. 96** **(50% vs. 65%)**	**<0.01**
c.*24G>A	A allele vs. GG	930 vs. 267 (78% vs. 22%)	158 vs. 45 (17% vs. 17%)	ns	316 vs. 109 (34% vs. 41%)	ns	482 vs. 142 (52% vs. 53%)	ns
	G allele vs. AA	834 vs. 363 (70% vs. 30%)	147 vs. 56 (18% vs. 15%)	ns	307 vs. 118 (37% vs. 33%)	ns	433 vs. 191 (52% vs. 53%)	ns
c.932G>A	G allele vs. AA	997 vs. 200 (83% vs. 17%)	167 vs. 36 (17% vs. 18%)	ns	347 vs. 78 (35% vs. 39%)	ns	518 vs. 106 (52% vs. 53%)	ns
	A allele vs. GG	767 vs. 430 (64% vs. 36%)	129 vs. 74 (17% vs. 17%)	ns	279 vs. 146 (39% vs. 34%)	ns	417 vs. 207 (54% vs. 48%)	ns

ns: non statistically significant; MACE: major adverse cardiovascular events; PCI: percutaneous coronary intervention; CABG: coronary artery bypass grafting; T allele = TT genotype + CT genotype; C allele = CC genotype + CT genotype; A allele = AA genotype + AG genotype; G allele = GG genotype + AG genotype; bold font indicates statistical significance.
